# Prevalence, patterns of multimorbidity, and its correlations with health-related quality of life in rural southwest China: a cross-sectional study

**DOI:** 10.3389/fmed.2025.1609831

**Published:** 2025-08-21

**Authors:** Na Xie, Huali Xiong, Xin Jiang

**Affiliations:** ^1^Chongqing General Hospital, Chongqing, China; ^2^Center for Mental Health of Rongchang District, Chongqing, China; ^3^Health Committee of Rongchang District, Chongqing, China

**Keywords:** prevalence, multimorbidity, multimorbidity pattern, health-related quality of life, correlation

## Abstract

**Background:**

The prevalence, patterns, and impact of multimorbidity on health-related quality of life (HRQoL) remain inadequately understood among rural populations in southwest China. This study seeks to fill this knowledge gap by systematically examining these aspects.

**Methods:**

Participants were recruited from the China Multi-Ethnic Cohort (CMEC) study. Incident cases of 13 chronic conditions were documented. Multimorbidity was defined as the presence of two or more chronic conditions in an individual. Principal component factor analysis (PCFA) was performed to identify patterns of multimorbidity. Tobit regression analysis and restricted cubic spline were employed to assess the correlation between multimorbidity patterns and HRQoL.

**Results:**

A total of 2,998 participants were enrolled, with a mean age of 50.65 years (SD = 11.99). The prevalence of multimorbidity was 48.50%. Four multimorbidity patterns were identified by PCFA: circulatory system pattern, digestive system pattern, metabolic syndrome pattern, and hepatobiliary system pattern. All four patterns were negatively correlated with HRQoL, as demonstrated by tobit regression analysis (*β* = −0.024, *β* = −0.020, *β* = −0.007, *β* = −0.018; all *p* < 0.001). Restricted cubic spline also demonstrated the negative correlation between different multimorbidity patterns and HRQoL, after adjusting for potential confounding factors. Subgroup analysis in different gender, age, and average yearly family total income also demonstrated these negative correlations.

**Conclusion:**

The prevalence of multimorbidity is relatively high in rural southwest China. Distinct multimorbidity patterns were correlated with poorer HRQoL. These findings enhance the understanding of multimorbidity patterns and may inform the development of tailored primary healthcare services.

## Introduction

Multimorbidity is commonly defined as the co-occurrence of multiple long-term health conditions in the same individual, requiring ongoing and diverse treatment (1). The prevalence of chronic diseases is rising partly due to improved health literacy and advancements in diagnostic technologies (2). The co-occurrence of multiple chronic conditions can result in complex interactions that not only significantly reduce the quality of life (3) but also impose a substantial economic burden on both families and society. Compared to individuals with a single chronic condition, patients with multimorbidity face higher risk of mortality (4) and increased healthcare costs, greater demand for medical services, and more frequent polypharmacy (5–7). Research on multimorbidity is crucial for advancing patient self-management, optimizing treatment outcomes, and facilitating the judicious allocation of healthcare facilities and resources, as well as informing policymaking and clinical guidance. Multimorbidity has emerged as a significant public health challenge in China, necessitating urgent attention and resolution.

Recent research shows increasing interest in multimorbidity among the middle-aged and elderly population. In the United States, over half of older adults suffer from three or more chronic conditions (8), while in China, the prevalence of multimorbidity among adults in China is 58.10% (9). Studies consistently link the number of chronic diseases to health-related quality of life (HRQoL) in the older adults (7) with systematic reviews confirming the inverse relationship between multimorbidity conditions and HRQoL (10, 11). While the body of literature on multimorbidity and HRQoL is growing, fewer studies explore multimorbidity patterns affecting HRQoL in the general population and the contributing factors. The main challenge in assessing multimorbidity risk lies in identifying and classifying its subtypes. Numerous studies have linked multimorbidity patterns to functional decline (12), depression (13), dosage frequency (14), and healthcare utilization (15). Common multimorbidity patterns are often identified in older population. Three major multimorbidity patterns were identified from 2015 waves of CHARLS study, including asthma/chronic lungs diseases, asthma, arthritis, or rheumatism/chronic lung diseases, dyslipidemia, hypertension, arthritis, or rheumatism/heart attack (5). Despite limited research on multimorbidity prevalence and patterns in the general population, a review summarized 51 studies and identified 407 profiles, and cardio-metabolic syndrome and mental health were more consistently observed in multimorbidity pattern studies (16).

HRQoL refers to a multidimensional concept that reflects individuals’ experiences across different cultures in relation to their goals, expectations, standards, concerns, and overall life circumstances. It provides a comprehensive evaluation of an individual’s disease burden or health status from physiological, psychological, and social perspectives. It has been extensively applied in assessing health conditions, evaluating the effectiveness of health services, and guiding the development of health service programs, thereby promoting the utilization of medical and healthcare resources. Although HRQoL loss associated with single chronic diseases has been well studied, research focusing on the impact of multimorbidity remains limited (17). Previous studies have identified a negative correlation between multiple chronic conditions and HRQoL (10). However, detailed knowledge about the influence of specific multimorbidity patterns on HRQoL is still lacking.

Previous studies have primarily focused on nationally representative samples of older Chinese adults. Recently, research has shifted from examining single diseases to multimorbidity in relation to health status. However, few studies have explored the correlation between multimorbidity patterns and HRQoL. This study was undertaken to examine the prevalence of multimorbidity and identify its common patterns in the general population of rural southwest China.

## Methods

### Study design and population

The data used in this study were obtained from the baseline survey of the China Multi-Ethnic Cohort Study (18), which was the largest cohort study conducted by Sichuan University in southwestern China from September 2018 to January 2019. Briefly in Rongchang region (19), participants were recruited via a three-stage stratified random sampling approach. Initially, four streets named Changyuan, Changzhou, Anfu, and Guangshun were randomly chosen from a total of 21 streets or towns. Subsequently, 10 villages were randomly selected from each of these streets. Finally, 50 to 80 individuals were randomly sampled in accordance with the age and sex distribution of the Rongchang population. Ethics approval was granted by the ethics committee of Sichuan University (No. K2016038), and all participants provided written informed consent.

The recruitment of participants in this study was guided by the following inclusion criteria: (1) age between 30 and 79 years at the time of the investigation; (2) having resided in Rongchang for at least 6 months; (3) Han ethnicity; (4) voluntarily participating in the survey, providing written informed consent, agreeing to provide biological samples, and committing to complete follow-up procedures; and (5) having no mental illness or cognitive disorders and possessing intact expressive abilities. Individuals were excluded if they met any of the following criteria: incomplete data regarding general characteristics, questionnaires, physical examinations, or blood biochemical tests. A total of 3,002 individuals were initially recruited during the baseline survey. After excluding those with missing data on general characteristics, the final analysis included 2,998 participants.

### Data collection

#### General characteristics

Age was expressed as mean and standard deviation and grouped into “30–39,” “40–49,” “50–59,” “60–69,” and “70–79” years. Gender was grouped into “males” and “females.” Marital status was grouped into “married/cohabiting” and “separated/divorced/widowed/unmarried.” Education level was grouped into “primary school or below,” “junior middle school,” and “high school or above.” Job status was grouped into “farmers,” “government employee,” “workers,” “sales & service staffs,” and “others.” Average yearly family total income was grouped into “<20,000 yuan,” “20,000–59,999 yuan,” “60,000–99,999 yuan,” and “≥100,000 yuan.”

#### Definition of healthy life factors

Non-smoking, moderate alcohol intake, healthy waist-to-hip ratio (WHR), adequate physical activity (PA), the dietary approaches to stop hypertension (DASH) score, and adequate sleep were considered six modifiable healthy lifestyle factors based on literature reviews (20, 21). Non-smoking was defined as participants who had never consumed any cigarette products. Moderate alcohol intake was defined as alcohol consumption within 1–14 g/day for females or 1–28 g/day for males (22) according to the self-reported alcohol consumption data. Healthy WHR was defined as <0.90 for males or <0.85 for females (20). WHR is calculated by dividing waist circumference by hip circumference. Physical activity levels, including work, transportation, household chores, and leisure-time activities, were assessed by metabolic equivalent tasks (METs) (23). Adequate PA was defined as having a total METs greater than 3,000 (19). A modified DASH diet score was calculated using a method adapted from prior study (24) with minor adjustments tailored to the CEMC dataset (25). The modified DASH score was constructed based on seven food categories: whole grains, fresh fruits, fresh vegetables, beans, dairy, red meat products, and sodium. Scores ranging from 1 to 5 were allocated according to the quintiles of average consumption for each food group. Specifically, participants in the highest quintile of intake for whole grains, fresh fruits, fresh vegetables, beans, and dairy were assigned a score of 5, while those in the lowest quintile of intake for red meat products and sodium received a score of 1. The overall DASH score for each participant was then calculated by summing the scores from all seven food groups (26). Based on the lowest tertile of the DASH score as the cutoff, participants were categorized into two groups: “<20” and “≥20.” Adequate sleep was defined as a sleep duration of 7–8 h/day (27).

#### Measurement of chronic diseases

A total of 21 chronic diseases or conditions were collected, including hypertension, diabetes, dyslipidemia, coronary heart disease, stroke, rheumatic heart diseases, pulmonary heart disease, pulmonary tuberculosis, chronic bronchitis/pulmonary emphysema, asthma, chronic hepatitis/cirrhosis, ulcers of the digestive tract, gastroenteritis, gallstones/cholecystitis, bone fracture, rheumatoid or arthritis, intervertebral disc disease, mental and psychological disorders, neurasthenia, brain injury, and cancers. The incidence rate of each chronic diseases or conditions is shown in Supplementary Table 1. To improve the robustness of the principal component factor analysis, chronic diseases with an incidence rate below 1% were excluded from the current study based on previous study (28).

Hypertension (29) was identified as having an average systolic blood pressure (SBP) of ≥140 mmHg or diastolic blood pressure (DBP) of ≥90 mmHg, based on three consecutive blood pressure measurements taken at 5-min intervals while the individual was at rest. It was also defined as having a prior physician diagnosis of hypertension or current use of blood pressure-lowering treatments (e.g., medications and exercise). Diabetes (30) was diagnosed when fasting plasma glucose (FPG) levels were above 7.00 mmol/L, or when there was a prior physician diagnosis of diabetes mellitus or the individual was on treatments to lower FPG levels (e.g., medications and exercise). Dyslipidemia was defined by the presence of any of the following four abnormalities (31): (1) total cholesterol ≥6.2 mmol/L; (2) triacylglycerol≥2.3 mmol/L; (3) low-density lipoprotein cholesterol≥4.1 mmol/L; (4) high-density lipoprotein cholesterol < 1.0 mmol/L.

Hypertension, diabetes, and dyslipidemia were verified through clinical or biometric methods, while other chronic diseases or conditions were self-reported by the participants. Coronary heart disease, rheumatic heart disease, and pulmonary heart disease were grouped into heart diseases. Multimorbidity was defined as the coexistence of multiple chronic conditions greater than or equal to two.

#### Health-related quality of life

HRQoL was measured using the European Five-Dimensional Five-level Health Scale (EQ-5D-5L) instruments (32), which enhances the ability to differentiate respondents’ health status and enhances objectivity in evaluating their subjective experiences. The scale consists of the EQ-5D health description system and the EQ-5D visual analogue scale. HRQoL serves as a multidimensional metric for assessing physical condition, mental functioning, social capability, and overall personal wellbeing, thereby reflecting the impact of diseases or physical and mental impairments on an individual’s overall health status. The EQ-5D health description system encompasses five dimensions: mobility, self-care, usual activities, pain/discomfort, and anxiety/depression. Each dimension is divided into five levels: no difficulty, slight difficulty, moderate difficulty, severe difficulty, and unable to complete/extreme difficulty. The five response levels (1–5) correspond to each dimension in sequence. Initially, a five-level health profile is generated (e.g., 55,555, indicating extreme difficulty or inability in all five dimensions), allowing the EQ-5D to represent a total of 3,125 (5^5^) distinct health states (33). Subsequently, the EQ-5D converts raw data into health utility values through a utility scoring system developed by Luo (34). The scale ranges from −0.391 to 1, where 0 corresponds to death and 1 denotes perfect health, and a health utility value below 0 indicates a health state deemed worse than death (35). The Chinese version of the EQ-5D-5L has been validated by Chinese researchers and has demonstrated satisfactory reliability and validity. The methodology for deriving EQ-5D-5L values is detailed in the Supplementary Tables 2, 3.

### Statistical analysis

Age and HRQoL were reported using mean and standard deviation, despite the absence of a normal distribution. General characteristics and healthy life factors were presented by constituent ratio (%), difference in HRQoL between binary variables was analyzed by Mann–Whitney U test, and differences among multivariate variables were analyzed by Kruskal–Wallis H test. Principal component factor analysis (PCFA) is one of the statistical approaches to identify non-random cluster patterns; we used PCFA with varimax rotation based on the same chronic conditions to z scores (25). After comprehensively considering the Kaiser–Meyer–Olkin measure of sample adequacy, Bartlett’s test of sphericity, eigenvalues, cumulative variance explained, scree plot, and interpretability into considerations, four major multimorbidity patterns were identified. For each multimorbidity pattern, factor scores were calculated for all participants by summing up the standardized chronic conditions weighted by their factor loadings. The multimorbidity patterns were assessed by a split-sample validation to support the robustness of the characteristics. Given the ability of tobit regression to effectively handle samples with limited data ranges and skewed distributions (such as health utility value was skewed and censored at 1) (28, 36). The influencing factors and multimorbidity patterns correlated with HRQoL were conducted by tobit regression.

The statistical significance of the tobit regression model was assessed using the likelihood ratio test. In addition, the model fit was evaluated based on the Akaike information criterion (AIC) and the Bayesian information criterion (BIC), with lower values indicating superior model performance. Restricted cubic spline was performed to examine the correlation between factor scores of different multimorbidity patterns and health utility values, after adjusting for potential confounding factors. To assess multicollinearity among the variables, we employed the variance inflation factor (VIF) and tolerance. A VIF value exceeding 5 was indicative of significant collinearity among the variables. In addition, tolerance was calculated as the reciprocal of the VIF (tolerance = 1/VIF). When the tolerance value was less than 0.2, it suggested the presence of substantial collinearity. Subgroup analyses in different gender, age, and average yearly family total income were also conducted to further assess the robustness of the results by tobit regression and restricted cubic spline. The statistical analysis was performed using R software (version 4.1.1), with a two-tailed *p*-value of less than 0.05 indicating a statistically significant difference.

## Results

### General characteristics and HRQoL of the participants

General characteristics and HRQoL of the participants are presented in Table 1. Among the 2,998 participants, the mean age was 50.65 (S.D. = 11.99) years and 50.03% of the participants were females. The mean health utility value was 0.95 (S.D. = 0.11), and 2020 of the participants was in the “perfect health” (health utility value = 1). Statistically significant differences were observed across various demographic variables, including gender, age, marital status, education level, job status, and average yearly family total income (all *P*<0.05).

**Table 1:** General characteristics and HRQoL of the participants.

Basic characteristic	*n*	%	HRQoL (Mean ± SD)	*Z*/*H*	*p*
Gender				*Z =* –4.115	<0.001
Male	1,498	49.97	0.96 ± 0.11		
Female	1,500	50.03	0.95 ± 0.12		
Age/year				*H =* 294.79	<0.001
30–39^#^	594	19.81	0.99 ± 0.03		
40–49	991	33.06	0.97 ± 0.09^*^		
50–59	623	20.78	0.95 ± 0.10^*^		
60–69	533	17.78	0.91 ± 0.16^*^		
70–79	257	8.57	0.89 ± 0.16^*^		
Marital status				*Z =* –3.712	<0.001
Married/cohabiting	2,705	90.23	0.96 ± 0.11		
Separated/divorced/widowed/unmarried	293	9.77	0.93 ± 0.14		
Education level				*H =* 78.669	<0.001
Primary school or below^#^	724	24.15	0.95 ± 0.12		
Junior middle school	840	28.02	0.93 ± 0.14^*^		
High school or above	1,434	47.83	0.97 ± 0.09^*^		
Job status				*H =* 114.012	<0.001
Farmers^#^	979	32.66	0.93 ± 0.14		
Government employee	317	10.57	0.98 ± 0.04^*^		
Worker	280	9.34	0.97 ± 0.09^*^		
Sales and Service staff	514	17.14	0.98 ± 0.04^*^		
Others	908	30.29	0.95 ± 0.11		
Average yearly family total income/Yuan				*H =* 92.486	<0.001
<20,000^#^	913	30.45	0.93 ± 0.14		
20,000–59,999	1,087	36.26	0.95 ± 0.12^*^		
60,000–99,999	524	17.48	0.97 ± 0.07^*^		
≥100,000	474	15.81	0.97 ± 0.06^*^		

Z. Mann–Whitney U test; H. Kruskal–Wallis H test. # Reference group; ^*^. Compared to the reference group, *P* < 0.05. HRQoL: health-related quality of life. HRQoL was expressed as mean and standard deviation.

### The healthy life factors and their difference in HRQoL

The healthy life factors and their difference in HRQoL are presented in Table 2. Among the 2,998 participants, 80.19% of the participants were non-smoking, 43.40% of the participants had a healthy WHR, 69.51% of the participants had adequate physical activities, 71.08% of the participants had a higher DASH score over 20, and 62.74% of the participants had adequate sleep. The HRQoL in non-smoking, healthy WHR, adequate PA, and adequate sleep are statistically significant difference (all *P*<0.05).

**Table 2:** Healthy life factors and their difference in HRQoL.

Healthy life factor	*n*	%	HRQoL (Mean ± SD)	*Z*	*P*
Smoking				−2.567	0.010
No	2,404	80.19	0.95 ± 0.12		
Yes	594	19.81	0.96 ± 0.08		
Moderate alcohol intake				−1.266	0.206
No	791	26.38	0.95 ± 0.11		
Yes	2,207	73.62	0.95 ± 0.11		
Healthy WHR				−4.525	<0.001
No	1,697	56.60	0.95 ± 0.11		
Yes	1,301	43.40	0.96 ± 0.11		
Adequate PA				−6.938	<0.001
No	914	30.49	0.93 ± 0.14		
Yes	2084	69.51	0.96 ± 0.09		
DASH score				−0.806	0.420
<20	867	28.92	0.94 ± 0.13		
≥20	2,131	71.08	0.96 ± 0.10		
Adequate sleep				−7.111	<0.001
No	1,117	37.26	0.93 ± 0.14		
Yes	1881	62.74	0.96 ± 0.09		

Z. Mann–Whitney U test. HRQoL: health-related quality of life; DASH: dietary approaches to stop hypertension; PA: physical activity; WHR: waist-to-hip ratio; S.D.: standard deviation. HRQoL was expressed as mean and standard deviation.

### Prevalence of multimorbidity, multimorbidity patterns, and its difference in HRQoL

The cases of each chronic disease or condition, both independently and in the context of multimorbidity, are presented in Table 3. A total of 732 participants (24.42%) were free from chronic disease or condition, while 812 participants (27.08%) were suffering from a single chronic disease or condition. The prevalence of multimorbidity was 48.50%. Participants suffering from rheumatoid arthritis, peptic ulcers, or heart disease exhibited a higher likelihood of experiencing multimorbidity, while those with chronic hepatitis/cirrhosis, dyslipidemia, and hypertension were less likely to have multimorbidity. Spearman’s correlation analysis found a negative correlation between the total number of chronic diseases or conditions and HRQoL (*r*_s_ = −0.349, *P*<0.001).

**Table 3:** Each chronic disease or condition independent of comorbidities (all cases) and by each chronic disease with multimorbidity.

Chronic disease/condition	All cases	Multimorbidity		
		*n*	%	Mean ± SD
Rheumatoid or arthritis	248	238	95.97	3.78 ± 1.63
Ulcers of the digestive tract	124	117	94.35	3.75 ± 1.77
Heart disease	123	116	94.31	4.27 ± 1.87
Chronic bronchitis/pulmonary emphysema	215	200	93.02	3.68 ± 1.77
Neurasthenia	99	92	92.93	4.17 ± 1.85
Diabetes	320	295	92.19	3.28 ± 1.40
Bone fracture	268	246	91.79	3.34 ± 1.60
Gallstones/cholecystitis	382	335	87.70	3.13 ± 1.59
Gastroenteritis	419	358	85.44	3.19 ± 1.64
Intervertebral disc disease	721	594	82.39	2.93 ± 1.54
Chronic hepatitis/cirrhosis	149	119	79.87	2.97 ± 1.76
Dyslipidemia	827	645	77.99	2.67 ± 1.45
Hypertension	1,197	925	77.28	2.60 ± 1.40

S.D., standard deviation.

Before performing the principal component factor analysis, we evaluated the suitability of the data by calculating the Kaiser–Meyer–Olkin (KMO) measure of sampling adequacy (KMO = 0.677, cutoff > 0.50) and performing Bartlett’s test of sphericity (*χ^2^* = 1396.26, *p* < 0.001). Both tests indicated significant correlations among the different chronic diseases or conditions, supporting the appropriateness of applying a factor analytic approach. The findings confirmed that the sample was appropriate for the generating distinct and reliable factors. Principal component factor analysis was conducted on the 13 chronic diseases or conditions, resulting in four factors with eigenvalues greater than 1 (1.904, 1.328, 1.099, and 1.050 for the first through fourth factors, respectively). These factors were extracted following varimax rotation with Kaiser normalization. Collectively, the four factors explained 41.39% of the cumulative variance (Supplementary Table 4). Furthermore, the inflection points observed in the scree plot supported the presence of four factors, further substantiating the appropriateness of the factor extraction (Supplementary Figure 1).

**Figure 1:**
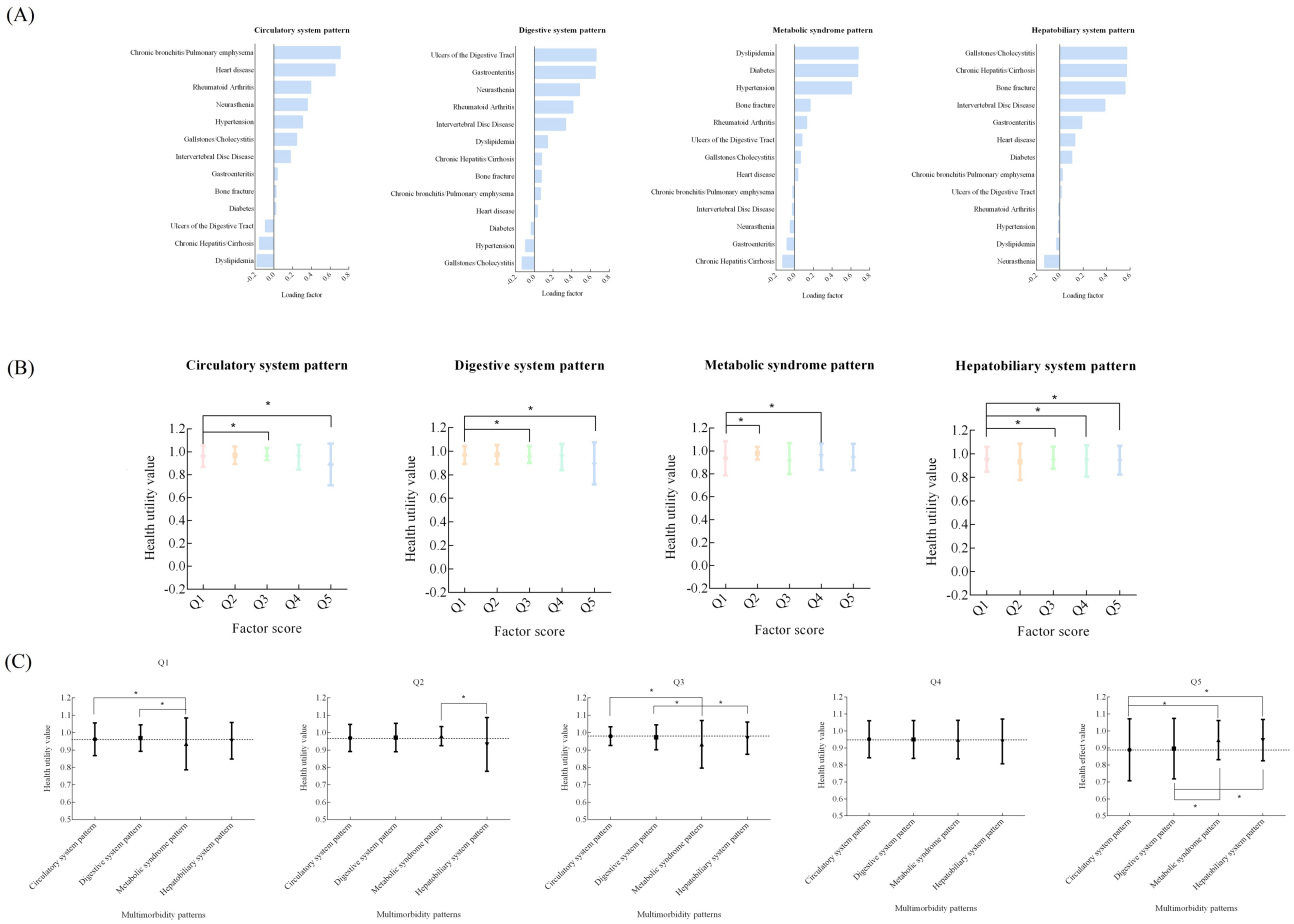
Exploratory factor analysis for 13 chronic diseases or conditions [**(A)** loading factor of four common patterns of multimorbidity; **(B)** the difference in health utility value from Q1 to Q5 in different multimorbidity pattern; **(C)** The difference of heath utility value in the same group of different multimorbidity pattern].

Figure 1A shows the multimorbidity pattern in the analysis, 4 common patterns of multimorbidity were observed, namely, circulatory system pattern (which predominantly consists of chronic bronchitis/pulmonary emphysema, heart disease, rheumatoid arthritis, neurasthenia, and hypertension), digestive system pattern (which predominantly consists of ulcers of the digestive tract, gastroenteritis, neurasthenia, rheumatoid arthritis, and intervertebral disc disease), metabolic syndrome pattern (which predominantly consists of dyslipidemia, diabetes, and hypertension), and hepatobiliary system pattern (which predominantly consists of gallstones/cholecystitis, chronic hepatitis/cirrhosis, bone fracture, and intervertebral disc disease). The loading factor of each multimorbidity pattern is shown in Supplementary Table 4.

Figure 1B shows the difference in health utility value from Q1 to Q5 in different multimorbidity patterns. Q5 always had a relatively lower HRQoL in circulatory system pattern, digestive system pattern, and hepatobiliary system pattern (*P*<0.05).

Figure 1C shows the difference of health utility value in the same group of different multimorbidity patterns. There were statistically significant difference among Q1, Q2, Q3, and Q5 groups (*P*<0.05). In Q5 group, circulatory system pattern had the lowest health utility value.

### The correlation between multimorbidity patterns and HRQoL by tobit regression and restricted cubic spline

The correlation between multimorbidity patterns and HRQoL by tobit regression is presented in Table 4. Multicollinearity diagnostics revealed the absence of multicollinearity among the variables. Specifically, the VIF values for all variable indicators were below 5, while the corresponding tolerance values exceeded 0.2 (Supplementary Table 5). The model fit of the tobit regression was evaluated using the −2 log likelihood ratio, AIC, and BIC. The corresponding results are presented in Supplementary Table 6. After adjusting for confounding factors, the adjusted model 2 demonstrated that the circulatory system pattern (*β* = −0.024, *P* < 0.001), digestive system pattern (*β* = −0.020, *P* < 0.001), metabolic syndrome pattern (*β* = −0.007, *P* < 0.001), and hepatobiliary system pattern (*β* = −0.018, *P* < 0.001) were negatively correlated with HRQoL. Restricted cubic spline also demonstrated the negative correlation between factor score of different multimorbidity patterns and health utility values, after adjusting for potential confounding factors, including basic characteristics and life factors (Supplementary Figure 2). Subgroup analysis in different gender, age, and average yearly family total income by tobit regression (Table 5) and restricted cubic spline analysis in different gender (Supplementary Figure 3), age (Supplementary Figure 4), and average yearly family total income (Supplementary Figure 5) also demonstrated those similar negative correlation.

**Table 4:** Association between multimorbidity patterns and HRQoL by Tobit regression, [*β*(95% *CI*)].

Model	Circulatory system pattern	Digestive system pattern	Metabolic syndrome pattern	Hepatobiliary system pattern
Crude Model	−0.032 (−0.035 — –0.028)^**^	−0.021 (−0.025 — –0.017)^**^	−0.009 (−0.013 — –0.006)^**^	−0.019 (−0.023 — –0.015)^**^
Adjusted Model 1	−0.025 (−0.029 — –0.021)^**^	−0.020 (−0.023 — –0.016)^**^	−0.005 (−0.009 — –0.001)^*^	−0.017 (−0.021 — –0.014)^**^
Adjusted Model 2	−0.024 (−0.028 — − 0.020)^**^	–0.020 (−0.023—-0.016)^**^	−0.007(−0.011 — –0.003)^**^	−0.018 (−0.021 — –0.014)^**^

**p < 0.01; *p < 0.05. Crude Model: No adjustments were made. Adjusted Model 1: adjusted for basic characteristics, including gender, age, marital status, education level, job status, and family total income. Adjusted Model 2: adjusted for basic characteristics and healthy life factor, including non-smoking, moderate alcohol intake, healthy waist-to-hip ratio, adequate physical activity, dietary approaches to stop hypertension scores, and adequate sleep.

**Table 5:** Subgroup tobit regression analysis among the correlation between multimorbidity patterns and HRQoL, [*β*(95% *CI*)].

Variable	Circulatory system pattern	Digestive system pattern	Metabolic syndrome pattern	Hepatobiliary system pattern
Gender				
Male	−0.024 (−0.029 — –0.019)^**^	−0.016 (−0.021 — –0.010)^**^	−0.002 (−0.007 — 0.003)^#^	−0.015 (−0.020 — –0.010)^**^
Female	−0.024 (−0.030 — –0.017)^**^	−0.023 (−0.028 — –0.018)^**^	−0.007 (−0.013 — –0.001)^*^	−0.020 (−0.026 — –0.014)^**^
Age/year				
30–39	−0.007 (−0.012 — –0.002)^**^	−0.010 (−0.014 — –0.007)^**^	0.005 (−0.002 — 0.008)^#^	−0.002 (−0.005 — 0.000)
40–49	−0.024 (−0.031 — –0.017)^**^	−0.012 (−0.018 — –0.007)^**^	0.003 (−0.003 — 0.008)^#^	−0.019 (−0.024 — –0.014)^**^
50–59	−0.019 (−0.028 — –0.011)^**^	−0.022 (−0.029 — –0.015)^**^	−0.003 (−0.010 — 0.005)^#^	−0.012 (−0.020 — –0.005)^**^
60–69	−0.028 (−0.038 — –0.017)^**^	−0.028 (−0.039 — –0.017)^**^	−0.021 (−0.033 — –0.009)^**^	−0.025 (−0.036 — –0.013)^**^
70–79	−0.027 (−0.039 — –0.014)^**^	−0.031 (−0.048—-0.013)^**^	−0.003 (−0.021 — 0.016)^#^	−0.029 (−0.048 — –0.009)^**^
Average yearly family total income/Yuan				
<20,000	−0.029 (−0.037 — –0.021)^**^	−0.027 (−0.035—-0.019)^**^	−0.003 (−0.011 — 0.006)^#^	−0.019 (−0.027 — –0.012)^**^
20,000–59,999	−0.019 (−0.026 — –0.012)^**^	−0.017 (−0.023—-0.010)^**^	−0.009 (−0.015 — –0.002)^*^	−0.020 (−0.027 — –0.014)^**^
60,000–99,999	−0.026 (−0.033 — –0.019)^**^	−0.016 (−0.022—-0.010)^**^	0.002 (−0.004 — 0.008)^#^	−0.011 (−0.017 — –0.006)^**^
≥100,000	−0.014 (−0.021 — –0.007)^**^	−0.011 (−0.017—-0.006)^**^	−0.007 (−0.013 — –0.002)^*^	−0.008 (−0.014 — –0.002)^**^

**p < 0.01; *p < 0.05; #*p* > 0.05.

## Discussion

To our knowledge, this is the first study to explore the prevalence of multimorbidity and examine its common patterns correlated with HRQoL among the general population in rural southwest China. The mean health utility value among the participants was 0.95, which was higher than the German population (0.90) (37) and South Australian population (0.91) (38). Differences in HRQoL may be attributed to the study populations, assessment methods, and levels of regional economic development. Multimorbidity occurred in 48.50% of the participants aged 30–79 years, which was slightly higher than the older Chinese aged over 50 years (42.40%) (39) and Japanese adult population (29.9%) (14). Although the impact of multiple chronic conditions has long been recognized in European health systems, chronic disease management in most countries still relies on single-disease control programs and fails to develop integrated health management for multiple chronic conditions (40). Therefore, it is crucial to identify the patterns of multiple chronic conditions and develop integrated interventions tailored to various combinations. This strategy aims to address the increasing prevalence of multimorbidity in China and improve overall health status. In the current study, four distinct patterns were identified through principal component factor analysis. These findings aim to meet the growing demand from epidemiologists, clinicians, and policymakers for a deeper understanding of the burden of multimorbidity, particularly its clinical and social implications. Moreover, health economists can apply these results in cost–benefit analyses to inform reimbursement decisions.

The current study demonstrated that individuals with rheumatoid or arthritis, ulcers of the digestive tract, and heart disease had the highest prevalence of multimorbidity, while individuals with chronic hepatitis/cirrhosis, dyslipidemia, and hypertension had the lowest multimorbidity prevalence. Yao (39) et al. reported the 91.60% of multimorbidity prevalence in heart problems, 77.50% in hypertension patients, and 75.90% in arthritis or rheumatism patients. Mino-Leon (41) et al. demonstrated 89.90% of multimorbidity prevalence in cardiac problems and 76.30% in hypertension patients. Marengoni (42) et al. found 97.90% of multimorbidity prevalence in subjects with heart failure, 95.20% in subjects with hip fracture, and 74.60% in subjects with chronic obstructive pulmonary disease. The difference in multimorbidity prevalence may be attributed to variations in disease identification, study populations, or methods of analyzing patterns of multimorbidity. In this study, we found a less reported higher prevalence of multimorbidity (95.97%) in rheumatoid or arthritis than a nationally representative older Chinese (75.90%) (39), which was consistent with a study in middle-aged and elderly populations in Xinjiang (43). Chongqing, located in southwestern China, is renowned for its hot pot, a globally recognized dish characterized by high oil and salt content. The substantial consumption of meat contributes to excessive purine intake. Although the humidity in Chongqing is relatively low, the local population believes that the spicy and numbing nature of hot pot induces sweating, which helps eliminate moisture from the body (19, 24).

The findings suggest that patterns of disease combinations can influence the adverse outcomes of HRQoL. Four common patterns of multimorbidity were identified through PCFA, namely, circulatory system pattern, digestive system pattern, metabolic syndrome pattern, and hepatobiliary system pattern, which were all significantly correlated with reduced HRQoL. Among the identified multimorbidity patterns in the current study, circulatory and metabolic system were replicable patterns in previous studies (16, 44). Differences in multimorbidity patterns may be attributed to variations in study design, sample selection, social demographic characteristics, eligible diseases, and statistical methods. The circulatory system pattern, which primarily includes chronic bronchitis/pulmonary emphysema and heart diseases, observed in current study, is consistent with the findings from previous research studies (39, 42). Previous studies have documented the increasing prevalence of heart diseases in China, accompanied by significant complications and mortality rates (45). Chronic lung disease often causes respiratory distress and persistent coughing, resulting in a reduced quality of life (46). The association between cardiovascular disease and pulmonary disease has been supported by epidemiological (47) and physiologic evidence (48). A Canadian study concluded strong negative correlations between these diseases and the physical components of HRQoL (49). The coexistence of cardiovascular and pulmonary diseases can impair energy levels and social functioning, leading to diminished psychosocial adaptation and resilience (50).

With regard to the metabolic syndrome pattern, including dyslipidemia, diabetes, and hypertension, previous studies have demonstrated that hypertension was commonly combined with diabetes (9). Hypertension combined with diabetes and dyslipidemia was a relatively common pattern of co-morbidity in China, both in the elderly and in young population (51). Evidence from the China Health and Retirement Longitudinal Study demonstrated a higher co-morbidity prevalence of 10.00% in older population aged over 60 years (52). Uncontrolled blood pressure, glucose, and lipid levels heighten the risk of complications including cardiovascular and cerebrovascular diseases, as well as chronic renal insufficiency, which seriously threaten physical state, mental health, and even quality of life (53). Cai (51) et al. found that the 5-year survival rate of patients with hypertension and diabetes combined with dyslipidemia was lower than that of the general population.

In this study, we identified a less commonly reported patterns of multimorbidity termed digestive system pattern, which included ulcers of the digestive tract, gastroenteritis, neurasthenia, rheumatoid arthritis, etc. Ulcers of the digestive tract and gastroenteritis frequently occurred together (54). Ulcers of the digestive tract had the second highest prevalence (94.35%) among all multimorbidities, while gastroenteritis showed a multimorbidity prevalence of 85.44%, which might explain their frequent coexistence. In addition, neurasthenia and rheumatoid or arthritis were also included in the digestive system pattern. Alprazolam, commonly used to treat neurasthenia, can cause gastric cramps, nausea, and vomiting (55). Furthermore, the use of non-steroidal anti-inflammatory drugs (NSAIDs) in the management of rheumatoid arthritis may predispose patients to gastrointestinal ulcers and bleeding (56).

The hepatobiliary system pattern, which primarily consisted of gallstones/cholecystitis, chronic hepatitis/cirrhosis, bone fracture, and intervertebral disc disease, has been rarely reported in previous studies with multimorbidity patterns. Studies have shown that gallstones were associated with sarcopenia (57), while sarcopenia was positively associated with falls or facture (58). Acute cholecystitis was considered as a complication following hip fracture (59). Furthermore, individuals with chronic liver disease face have a heightened risk of developing osteoporosis, with fractures considered potential complications of this condition (60). The interaction of diseases between hepatobiliary system and fracture may partly explain the potential mechanism.

Although the Chinese government made an explicitly prioritization on development of rural healthcare and the launch of initiatives such as the New Rural Cooperative Medical System, rural healthcare continued to face serious challenges. Individuals living rural areas lack access to high-quality healthcare services, including accurate diagnosis, thorough physical examination, and timely treatment (61), which may result in lower self-awareness for health status. Primary healthcare is facilitated through public health and hospital services. Given that HRQoL is strongly influenced by physical wellbeing, psychological functioning, social capability, and overall personal status, these findings suggest that strategies aimed at improving quality of life outcomes are the most promising. The decline in HRQoL represents a critical clinical endpoint in the management of chronic diseases and multimorbidity. The results of the current study can help identify patients with multimorbidity for better resource allocation. Recognizing these patterns can be useful in disease categorization, management, reducing disease burden, and enhance patient health quality.

Therefore, we conclude that multimorbidity is even more critical than initially thought. The identification of multimorbidity patterns suggests that, in chronic disease management, policymakers and clinicians should not focus solely on individual chronic diseases. Instead, they should identify high-risk groups based on multimorbidity patterns and prioritize interventions accordingly. It is recommended to classify individuals with different chronic diseases for early assessment according to their risk of developing multimorbidity patterns and provide individualized management. For example, patients with rheumatoid arthritis should be regularly screened for pre-existing gastric or digestive diseases. In subgroup analyses, we observed that not all metabolic syndrome patterns exhibited statistically significant negative correlations with HRQoL, regardless of gender, age, or average yearly family total income. This finding underscores the complex nature of metabolic comorbidities. In addition, restricted cubic spline analysis among individuals aged 70–79 years showed that the digestive system, metabolic syndrome, and hepatobiliary system patterns each demonstrated non-linear “S” negative correlations with HRQoL. These results further emphasize the complexity of comorbid conditions in older adults.

The findings from the current research remain highly relevant, providing crucial insights for informing public health policies and guiding clinical interventions. This study has several notable strengths. First, it utilizes a large and representative sample drawn from the general population in southwest China. Second, the application of the EQ-5D-5L with the Chinese value set enhances the precision of our assessment, consistent with guidelines recommending country-specific evaluations. However, the potential limitations also should be acknowledged. First, we investigated only the prevalence of 13 common chronic diseases, and some diseases or conditions were underrepresented, potentially leading to an underestimation of the co-morbidity rate. Second, most of chronic conditions were self-reported by participants, which may introduce recall bias and potential misclassification errors as individuals may not accurately remember or describe their medical histories or conditions. Third, prevalent diseases or conditions with high prevalence, such as stroke, asthma, cancer, vision problems, hearing problems, and neurodegenerative diseases, were excluded. This exclusion might have resulted in underestimation attributable to recall bias or inaccuracies. Nonetheless, a systematic review reported a weak positive correlation between the number of chronic diseases included and multimorbidity, although this correlation was not statistically significant (*p* = 0.11) (62). Finally, detailed information on disease severity or duration time was not included in the current study.

## Conclusion

The current study confirmed the prevalence and patterns of multimorbidity among residents in rural areas of southwest China and highlighted the importance of shifting from single disease treatment to multimorbidity prevention and management for rural adults in southwest China. Continued efforts to improve our understanding of multimorbidity patterns will facilitate the development of innovative primary care models and comprehensive home-based care models tailored to the specific needs of individuals with diverse multimorbidity patterns. The goal of improving health related quality of life can only be achieved by fully recognizing the heterogeneity of multimorbidity patterns. In other regions, effective prevention and control of chronic diseases require a comprehensive understanding of these patterns. Specifically, it is crucial to elucidate the distinct characteristics of multimorbidity across demographic groups, including gender and age. Based on this knowledge, targeted strategies for prevention, control, and treatment can be devised. Furthermore, rational allocation of health resources and greater emphasis on primary healthcare are essential to enhance the effectiveness of multimorbidity management.

## Data Availability

The original contributions presented in the study are included in the article/Supplementary material, further inquiries can be directed to the corresponding author/s.
