# Supplementary figures and images for: Prevalence, patterns of multimorbidity, and its correlations with health-related quality of life in rural southwest China: a cross-sectional study
Source: Front Med (Lausanne). 2025 Aug 21;12:1609831. doi: 10.3389/fmed.2025.1609831 (PMC12408566; doi:10.3389/fmed.2025.1609831)

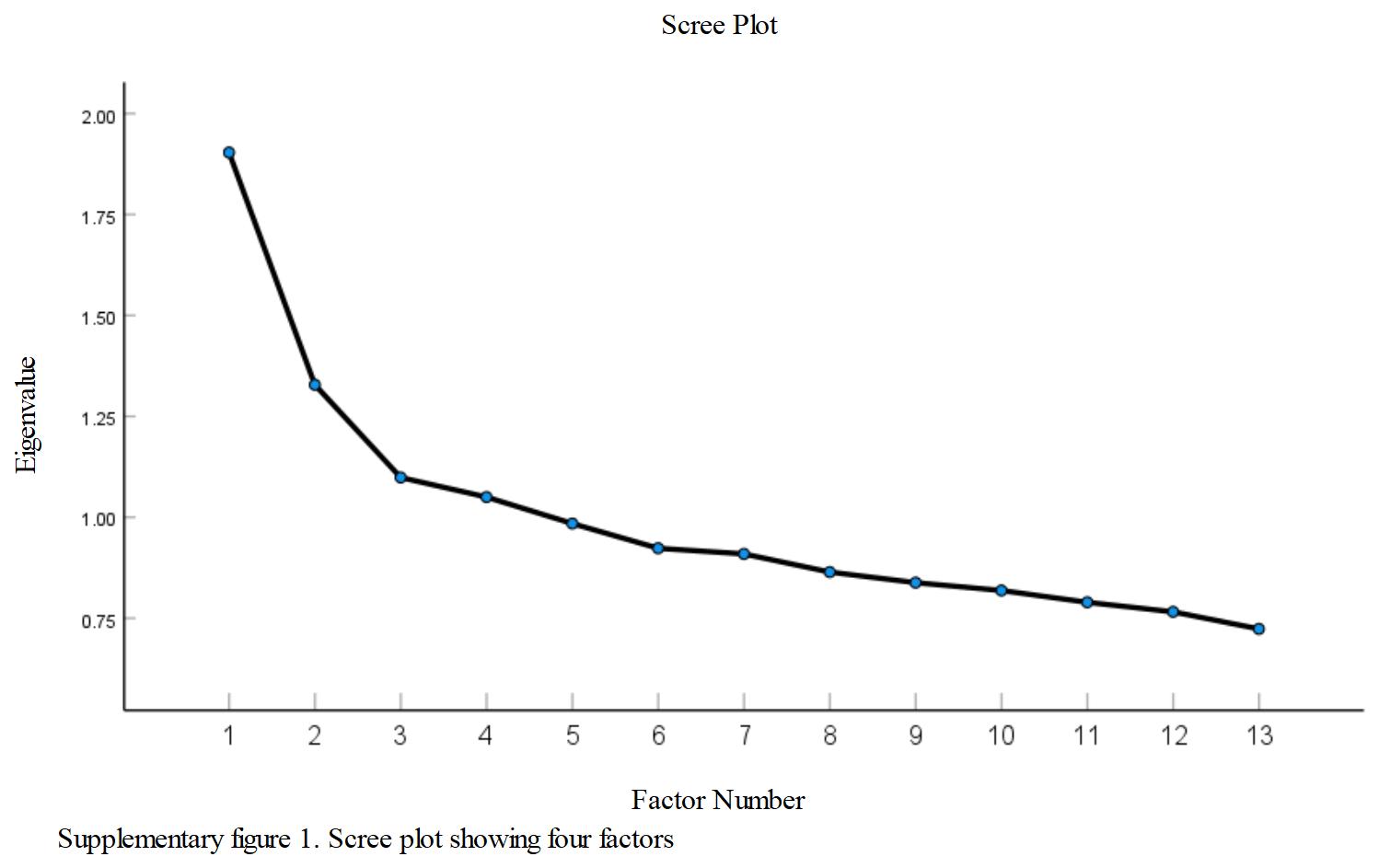

Supplement: Supplementary file 8 [file Image_1.JPEG]

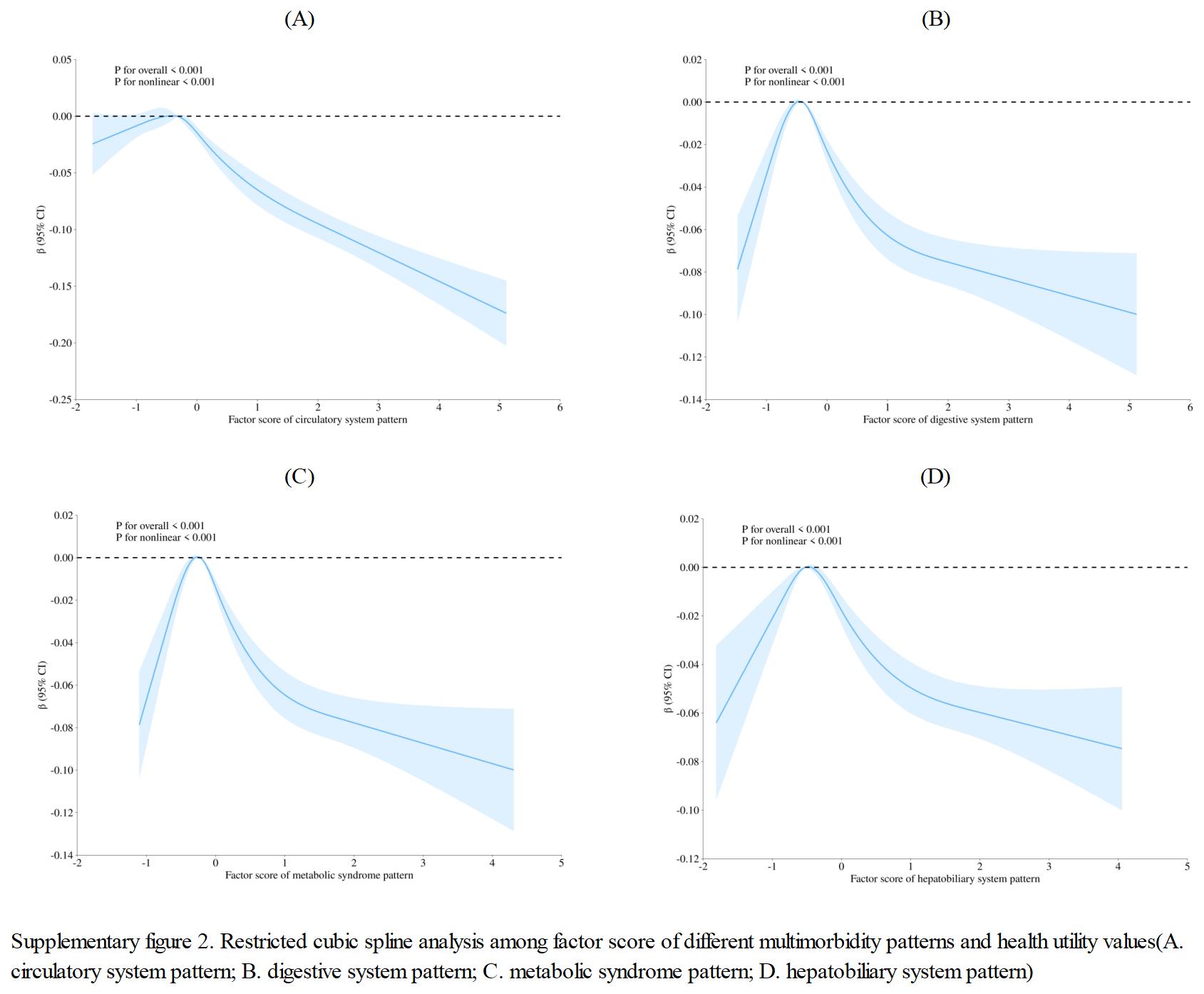

Supplement: Supplementary file 9 [file Image_2.JPEG]

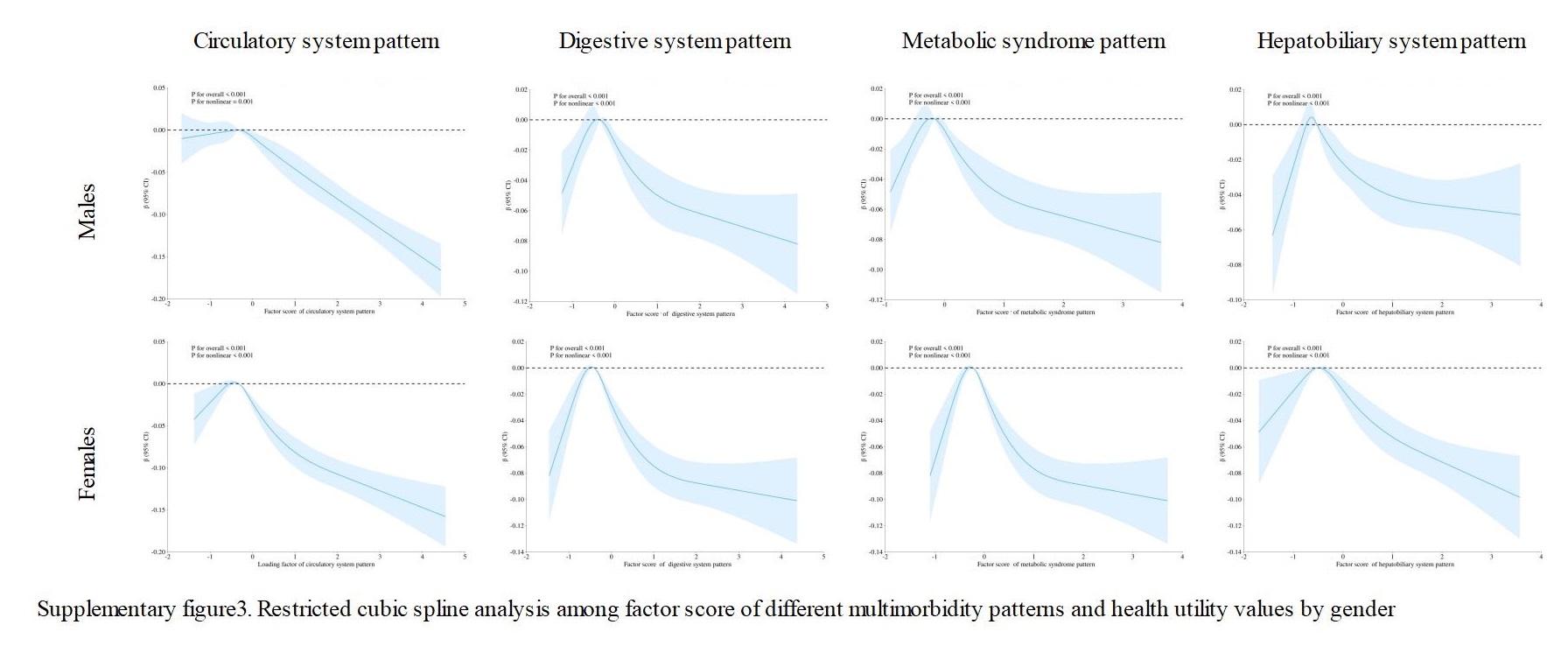

Supplement: Supplementary file 10 [file Image_3.JPEG]

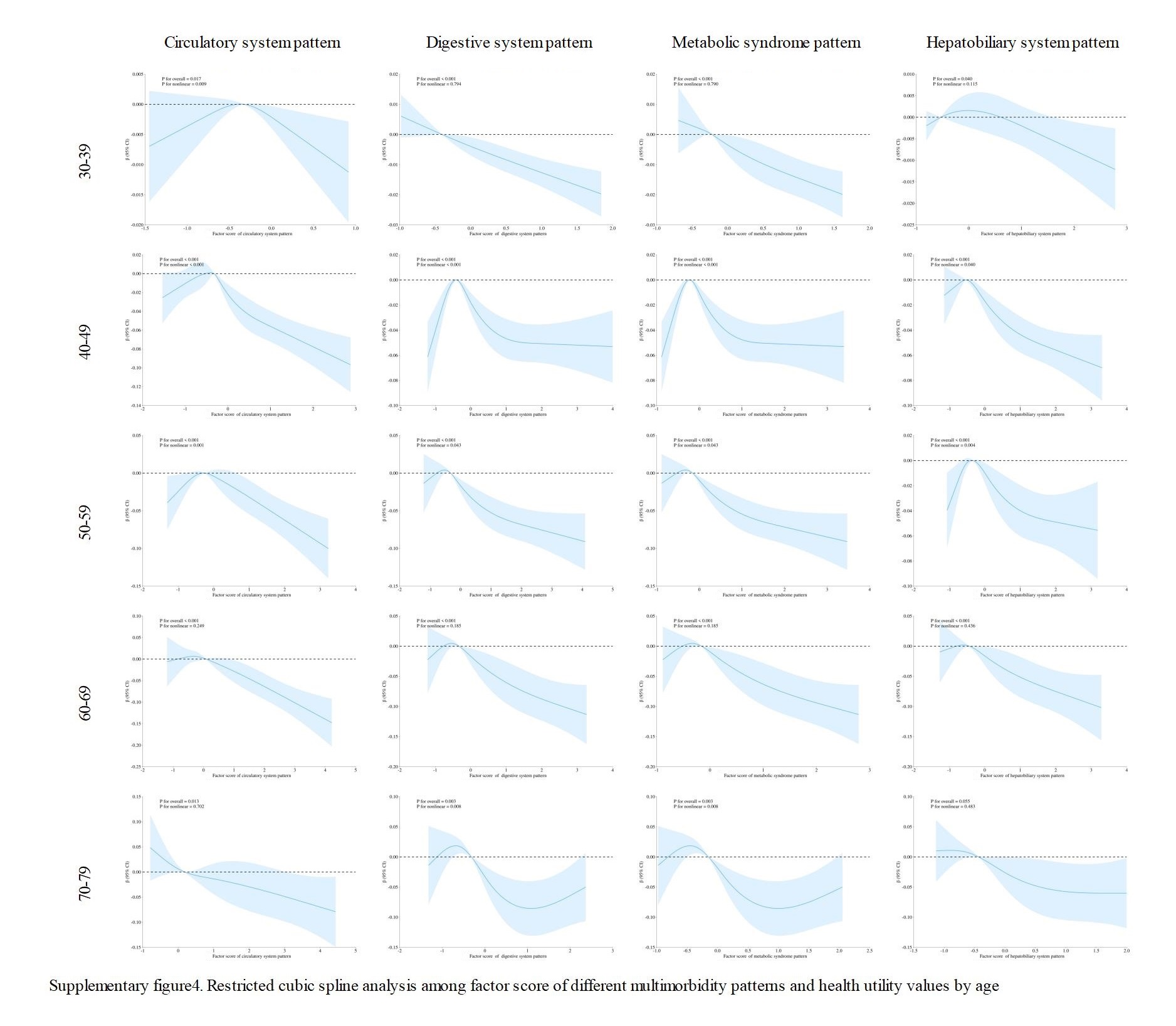

Supplement: Supplementary file 11 [file Image_4.JPEG]

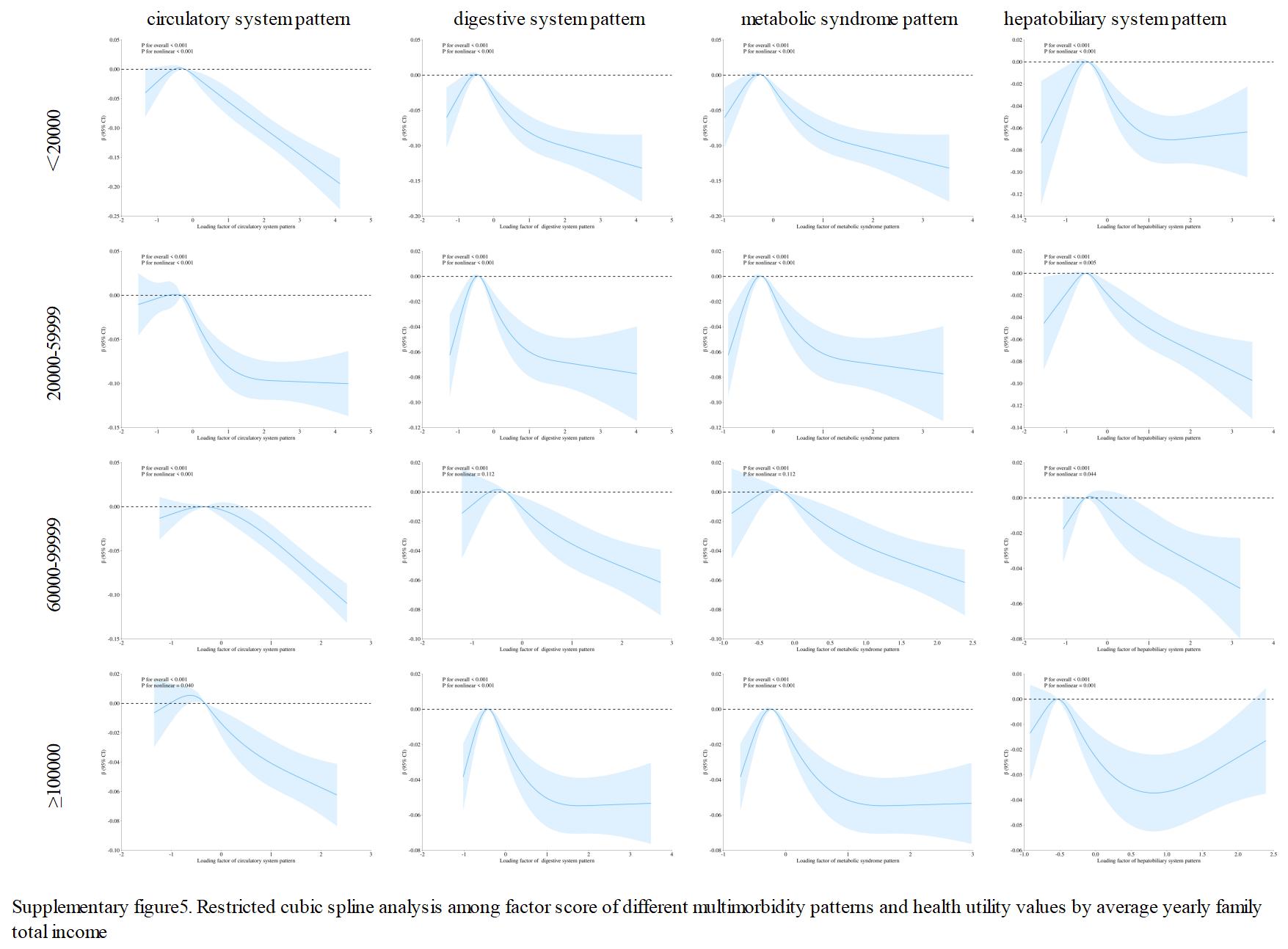

Supplement: Supplementary file 12 [file Image_5.JPEG]
